# Monotreme middle ear is not primitive for Mammalia

**DOI:** 10.1093/nsr/nwab131

**Published:** 2021-07-23

**Authors:** Jin Meng, Fangyuan Mao

**Affiliations:** Division of Paleontology, American Museum of Natural History, USA; Earth and Environmental Sciences, Graduate Center, City University of New York, USA; Division of Paleontology, American Museum of Natural History, USA; Key Laboratory of Evolutionary Systematics of Vertebrates, Institute of Vertebrate Paleontology and Paleoanthropology, Chinese Academy of Sciences, China; CAS Center for Excellence in Life and Paleoenvironment, China

The study of the evolution of the mammalian middle ear has been fueled by continuous discoveries of Mesozoic fossils in the last two decades. Wang *et al.* [1] recently reported a specimen of *Vilevolodon diplomylos* [2] that adds to the increasing knowledge about the auditory apparatus of ‘haramiyidans’, an extinct Mesozoic group of mammaliaforms. The authors hypothesized that a middle ear with a monotreme-like incus and malleus and incudomallear articulation was primitive for mammals, which challenges the convention that the monotreme middle ear is specialized [3] or autapomorphic [4] in mammals. We raise concerns about terminology (see Supplementary Data) and identification of the incus presented by Wang *et al.* and show that their analysis does not support their preferred hypothesis but favors the alternative, which is consistent with Mao *et al.*’s hypothesis that the braced hinge joint is primitive for several lineages within Mammalia [5].

Wang *et al.* presented some valuable interpretations on previously known but still poorly understood auditory bones, such as the surangular and ectotympanic, in haramiyidans. Because these subjects have been extensively treated [2,5–8], we focus our discussion on the new evidence that Wang *et al.* provided about the incus and malleus, from which they drew their conclusion. The authors claimed that in the specimen (IMMNH-PV01699) the ossicular chain is ‘well-preserved and in near-life position’ and that the incus and incudomallear articulation were monotreme-like. We noted, however, that these structures differ notably from those in the holotype of *V. diplomylos*, which was portraited as not to be monotreme-like [2]. Wang *et al.* thought that the incus ‘resembles’ and ‘has a similar outline’ to those of the *Jeholbaatar* [9] and *Arboroharamiya allinhopsoni* [6–8]. The ‘incus’ of *Jeholbaatar* has been shown to be part of the malleus by new evidence [5], as noted by Wang *et al.* The only known unequivocal incus of euharamiyidans is from *Arboroharamiya*, a sister taxon of *V. diplomylos*, that has been repeatedly described as having a convex and bulbous body and a long stapedial process with a lenticular process, like that of therians [6–8]. The so-called ‘incus’ in *Qishou*, as re-interpreted by Wang *et al.* (see Extended Data Fig. 6 in ref. [1]), is a misinterpretation—it is part of the element with debatable identity (Fig. 1k–m). Why is the incus identified in IMMNH-PV01699 so different from that of the holotype and its sister taxon but similar to monotremes? The possibility that it is a non-incus structure, as those interpreted in *Jeholbaatar* and *Qishou*, cannot be ruled out. This could explain why both sets of the incus and malleus ‘were moved to that degree from their position in life and yet remain well preserved’ [1]. It is uncertain whether Wang *et al.*’s computerized tomography (CT) data with a voxel size of 32.7 μm could secure the identities of the incus and malleus; a CT-slice showing the suture between them, as we did in Fig. 1, would settle the issue. We could not verify this because the digital data were not yet available.

**Figure 1.**
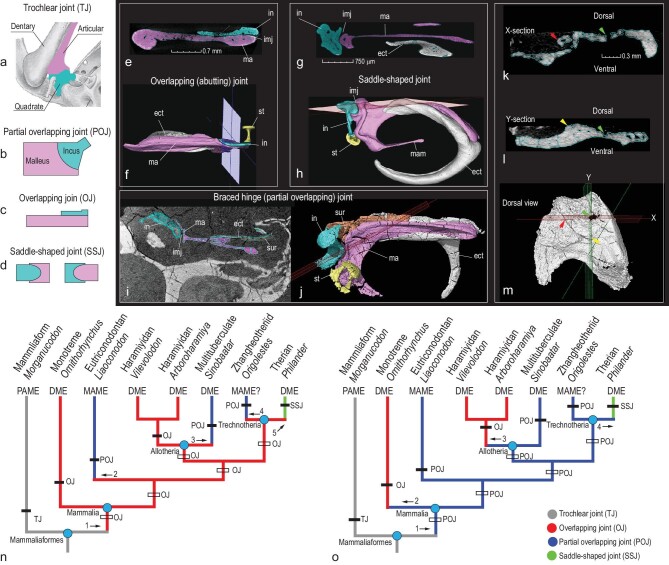
Types of incudomallear articulation and two hypotheses for the evolution of the mammalian middle ear. (a–d) Diagrams showing the trochlear, partial overlapping, overlapping and saddle-shaped incudomallear joints. (e–m) Computerized tomography images showing middle ear structures discussed in the text (see Supplementary Data for sources and abbreviations of the images). (n) Hypothesis preferred by Wang *et al.* [1], in which the monotreme-like overlapping incudomallear joint is primitive for Mammalia. (o) Alternative hypothesis overlooked by the authors, in which the braced hinge joint (= POJ) is primitive for Mammalia. Taxa, tree topology and optimized character distributions are from the original study (see Fig. 3 in ref. [1]). We added the empty bars, arrows and associated labels to visualize the evolutionary changes within the phylogeny. The comparison shows that the overlooked hypothesis (o) is more parsimonious than (n), which supports the existing hypothesis [6] but rejects the one that states that the monotreme middle ear is primitive for Mammalia.

Based on their identification of IMMNH-PV01699, Wang *et al.* concluded that optimization of five incudomallear characters in its phylogeny (Fig. 1n) ‘supports the overlapping joint as primitive for Mammalia. The partial overlapping joint is derived from the overlapping joint (and not vice versa) by the caudal shift of the incus with regard to the malleus.’ This contradicts the hypothesis that the braced hinge joint (= partial overlapping joint, POJ; Fig. 1b, i and j) is potentially primitive for mammals [5]. We noted that in non-monotreme mammals the five incudomallear characters were coded only in *Vilevolodon*, *Qishou* and *Arboroharamiya* [1]. However, the incus was not preserved in *Qishou* [8,10] (Fig. 1m) and the so-called malleus is subject to interpretation [1,5,10]. The two species of *Arboroharamiya* were coded as having a plate-like incus, which is factually untrue, as mentioned above. *Sinobaatar* was coded as ‘?’ for the five characters, although its well-preserved malleus and incus [5] have been illustrated in Wang *et al.*’s Fig. 3. To our knowledge, except for the purported monotreme-like incudomallear joint in IMMNH-PV01699, there is no convincing evidence for such a joint in any non-monotreme mammals and their relatives [1,5]. Of the five incudomallear characters, none showed up as a synapomorphy at any major node (clade) in the consensus tree (see Supplementary Information of ref. [1]). With an unstated method, Wang *et al.* managed to optimize the five characters and map the four types of joints (Fig. 1n) on the simplified consensus tree to show their preferred hypothesis.

Under their hypothesis (Fig. 1n), the first evolutionary step would be from the trochlear joint (TJ) in nonmammalian cynodonts to the monotreme ‘overlapping joint’ (OJ) in Mammalia. This step requires several abrupt changes (transformation through the POJ was deemed impossible by Wang *et al.*): the incus becoming a flat platelet, complete loss of the synovial joint, and the incudomallear complex transforming to a nearly horizontal position with the incus shifting to the dorsal side of the malleus. As known in some developmental studies, the vertical orientation of the ectotympanic in ontogeny was recognized as primitive in mammals [4] and therians [11] because the angular bone in nonmammalian cynodonts was vertically positioned. In the development of echidna the ectotympanic and malleus perform a ‘flipping’ from their original vertical position to horizontal orientation in adults [12]. The flat incus lying medial to the malleus and a horizontal ectotympanic were considered autapomorphic for monotremes [4]. These studies do not support Wang *et al.*’s hypothesis. In addition, this evolutionary step requires direct change from the ‘postdentary attached middle ear’ to the full ‘detached middle ear’ at Mammalia and demands independent regain of the ossified Meckel's cartilage (OMC) in adults of zhangheotheriids and eutriconodontans; this implies functional re-association of the auditory bones (hearing) with mastication at least in eutriconodontans. These changes are supported by no fossil or developmental evidence. Within Mammalia, two evolutionary steps from the OJ to POJ took place independently at eutriconodontans and multituberculates; furthermore, the OJ at Trechnotheria would have to give rise either to POJ, which then evolved into the saddle-shaped joint (SSJ) (Fig. 1d, g and h), or to the POJ and SSJ respectively; either of the two processes involves at least two evolutionary steps. Thus, at least four evolutionary steps are required within Mammalia (Fig. 1n).

It appears that Wang *et al.* have overlooked a better supported result of their optimization: the POJ is primitive for Mammalia, as we present in Fig. 1o. Under this alternative hypothesis, the evolutionary change from the nonmammalian cynodont TJ to the mammalian POJ would be simple because the incus and malleus retain the trochlear joint, the incus is largely caudal to the malleus and the auditory bones are positioned nearly vertically. Further, this evolutionary step requires neither full detachment of the auditory bones at Mammalia nor regain of the OMC in adults of zhangheotheriids and eutriconodontans. Within Mammalia there are only three evolutionary steps: two independent evolutions of the OJ at monotremes and haramiyidans, respectively, and one from the POJ to SSJ within Trechnotheria.

Wang *et al.* postulated their hypothesis based on the less-supported result of their analysis. Under the rule of parsimony, that hypothesis (Fig. 1n) should be falsified because it requires at least five evolutionary steps in the mammalian middle ear evolution. In contrast, their analysis corroborates the alternative (Fig. 1o) that needs only four steps, which supports the existing hypothesis [5]. Wang *et al.*’s conclusion that the monotreme-like middle ear is primitive for Mammalia is misleading.

## Supplementary Material

nwab131_Supplemental_File
