# Supplementary material for: Monotreme middle ear is not primitive for Mammalia
Source: Natl Sci Rev. 2021 Jul 23;8(10):nwab131. doi: 10.1093/nsr/nwab131 (PMC8566185; doi:10.1093/nsr/nwab131)
Supplement: nwab131_Supplemental_File [file nwab131_supplemental_file.docx]

**Supplementary Information for**

**Monotreme middle ear is not primitive for Mammalia**

Jin Meng^1, 2^* and Fangyuan Mao^3, 4, 1^

^1^ Division of Paleontology, American Museum of Natural History, USA.

^2^ Earth and Environmental Sciences, Graduate Center, City University of New York, USA.

^3^ Key Laboratory of Evolutionary Systematics of Vertebrates, Institute of Vertebrate Paleontology and Paleoanthropology, Chinese Academy of Sciences, China.

^4^ CAS Center for Excellence in Life and Paleoenvironment, China.

**Terminology issues**

Wang et al.^1^ started their work in promoting a set of terms to replace long-standing ones used for auditory bones with the intention to make them ‘descriptive’. They replaced, for instance, the ‘definitive mammalian middle ear’ (DMME)^13,14^ with ‘detached middle ear’ (DME)^15^. We agree that the DMME is literally imperfect, not because of its descriptive insufficiency but because of its bearing on phylogeny, which is rooted in an intricately historical context when phylogenetically defined Mammalia^16^ was not introduced to the study of mammalian evolution. In a later study, Allin and Hopson^14^ provided a clear definition for the DMME: “The configuration in which the angular, articular plus prearticular, and quadrate are strictly auditory structures, fully divorced from the feeding apparatus [and renamed tympanic, malleus, and incus].” In contrast, Wang et al. defined the DME as the configuration in which “the postdentary bones lack a bony or cartilaginous attachment to the mandible and have an exclusive auditory function”. Compared to the definition for the DMME^14^, this DME definition is poorly formulated because it does not include the incus (quadrate). Moreover, by definition, the angular (ectotympanic), surangular, and even the Meckel’s cartilage (as shown in fig. 1 in ref. 1) should be considered as parts of the middle ear, which is against Wang et al.’s will to “restrict middle ear ossicles to the bones in extant mammals that are suspended within the middle ear” (e.g., the stapes, malleus and incus only).

The authors used a mixture of terms in their study, including ‘auditory elements’, ‘auditory apparatus’, ‘middle ear’, ‘ossicular chain’, and ‘auditory bones’, while they disliked ‘auditory bones’ for a good reason. The auditory bones were specified as the stapes, malleus, incus, ectotympanic, and surangular in a Cretaceous mammal, *Origolestes*^17^. Wang et al. rightfully argued that other bones, such as the petrosal and squamosal, may function for hearing as well, an issue that has been extensively discussed elsewhere^6^. These authors preferred to use terms such as ‘auditory elements’ in their study, but their preference does not improve the terminology. Are the petrosal and squamosal auditory elements? Are the stapedius muscle, the tensor tympani muscle, and the tympanic membrane auditory elements? They are.

Moreover, Wang et al.’s new terms such as ‘postdentary attached middle ear’ (PAME) do not have clear definition and are intrinsically contradictory. The postdentary bones (articular, prearticular, angular, and surangular)^14^ comprise the middle ear, apart from the jaw joint, and are not an attachment tool for the middle ear. The PAME was coined to replace the ‘mandibular middle ear’. As already stated elsewhere, the typical mandibular middle ear “is represented by the middle ear of *Morganucodon* (Kermack et al., 1981) and was defined as the middle ear in which the articular-prearticular-angular complex is still directly attached to the dentary bone and the incus is in braced articulation with bones of the cranium; these bones have a dual-function of jaw suspension and hearing.”^6^ Why should we replace a well-defined and widely used term with a poorly defined and ill-phrased new one? We argue that terms such as “dentary attached middle ear” or “postdentary middle ear” may better describe the configuration termed the PAME, but we do not see the necessary to use these terms just because we think they sound better.

It would be better for science to keep coining new terms at the minimum, and if new terms have to be coined, the rationale for doing so should be clear and consistently applied throughout while recognizing the history of science and the rule of priority. New terms are necessary for novel structures, but it only worsens the already complicated anatomic terminology if one replaces existing terms with ill-defined new ones.

**Sources of figures**

**Fig. 1a** **-** Drawing of the mandibular middle ear of *Morganucodon*, showing the trochlear incudomallear joint (TJ). The figure is modified from Allin and Hopson (1992)^14^ with permission of reuse from Springer Nature.

**Fig. 1b.** - Diagram showing the braced hinge (partial overlapping) joint in cross-sectional view, which is based on Fig. 1i and 1j. In this type of joint, the incus is largely caudal to the malleus but laterally braced by a bony lip of the malleus. The figure is adopted from Mao et al. (2020)^5^.

**Fig. 1c.** - Diagram showing the overlapping (abutting) joint in cross-sectional view, which is based on **e** and **f**. In this type of joint, the incus is dorsal to the malleus and the contact between the incus and malleus is nearly flat.

**Fig. 1d.** - Diagram showing the saddle-shaped incudomallear joint in lateral (left) and dorsal (right) views, which are based on **g** and **h**, respectively. In this type of joint, the incus is caudal to the malleus.

**Fig. 1e-f. -** A CT-slice (e) that runs through the CT rendered ossicles of the monotreme *Tachyglossus* (f). The CT-scan has a voxel size of 8.153µm. The figure is modified from Mao et al. (2020)^5^.

**Fig. 1g-h. -** A CT-slice (g) that runs through the CT rendered ossicles of the marsupial *Didelphis* (g). The CT-scan has a voxel size of 6.611µm. The figure is modified from Mao et al. (2020)^5^.

**Fig.1i-j. -** A CT-slice (i) that runs through the CT rendered ossicles of the symmetrodontan *Origolestes*^5^. The CT-scan has a voxel size of 7.474µm. The figure is modified from Mao et al. (2020)^5^.

**Fig.1k-m. -** Two CT-slices (k and l) that run through the CT rendered element originally identified as the ectotympanic (**m**) of the euharamiyidan *Qishou*^6,8^. This element was re-interpreted as to be the malleus plus the incus by Wang et al.^1^. However, our CT-images show that Wang et al. has misinterpreted the structure because there is only one element, not two, regardless its identification; it is certain that the incus remains unknown in *Qishou*. The CT-scan has a voxel size of 10.2µm. The colored arrows in k and l correspond to the same reference point in m.

**Fig. 1 Abbreviations:** **ect,** ectotympanic; **imj,** incudomallear joint; **in,** incus; **ma,** malleus; **mam**, manubrium of the malleus; **st,** stapes; **sur**, surangular.

**References**

13 Allin, E. F. Evolution of the mammalian middle ear. *J Morphol* **147**, 403-437 (1975).

14 Allin, E. F. & Hopson, J. A. in *The evolutionary biology of hearing* (eds D.B. Webster, A.N. Popper, & R.R. Fay) 587-614 (Springer, 1992).

15 Harper, T. & Rougier, G. Petrosal morphology and cochlear function in Mesozoic stem therians. *PLoS one* **14**, e0209457 (2019).

16 Rowe, T., 1988. Definition, diagnosis, and origin of Mammalia. Journal of vertebrate Paleontology, 8(3), pp.241-264.

17 Mao, F. *et al.* Integrated hearing and chewing modules decoupled in a Cretaceous stem therian mammal. *Science* **367**, 305-308 (2020).
